# Supplementary material for: Investigating the biochemical variations in onion leaves due to purple blotch disease and its management through induced resistance
Source: PLoS One. 2025 May 9;20(5):e0323067. doi: 10.1371/journal.pone.0323067 (PMC12063818; doi:10.1371/journal.pone.0323067)
Supplement: Attachment — Submitted filename: Response to Reviewers_PONE-D-24-22600.docx [file pone.0323067.s003.docx]

Dr. Nasir Ahmed Rajput

Associate Professor

Department of Plant Pathology,

University of Agriculture, Faisalabad Pakistan

Email: [nasirrajput81@gmail.com](mailto:nasirrajput81@gmail.com); [nasir.ahmed@uaf.edu.pk](mailto:rashid.iqbal@iub.edu.pk)

**Date: August 21, 2024**

**Dear Muhammad Nauman Ahmad, PhD**

**Academic Editor**

**PLOS ONE**

Many thanks to you and the both reviewers for your comments on our MS!

We have carefully revised our MS as requested by the reviewer #1 and #2 and formatted the MS file following the online instructions.

Please see the point-by-point response to the reviewers and the revised MS file for the details. We hope this revision is now satisfactory for publication.

Yours sincerely,

**Nasir Ahmed Rajput**

|  | Dear Dr. Rajput,  Thank you for submitting your manuscript to PLOS ONE. After careful consideration, we feel that it has merit but does not fully meet PLOS ONE’s publication criteria as it currently stands. Therefore, we invite you to submit a revised version of the manuscript that addresses the points raised during the review process.  Please submit your revised manuscript by Aug 26 2024 11:59PM. If you will need more time than this to complete your revisions, please reply to this message or contact the journal office at plosone@plos.org. When you're ready to submit your revision, log on to https://www.editorialmanager.com/pone/ and select the 'Submissions Needing Revision' folder to locate your manuscript file.  Please include the following items when submitting your revised manuscript:   - A rebuttal letter that responds to each point raised by the academic editor and reviewer(s). You should upload this letter as a separate file labeled 'Response to Reviewers'. - A marked-up copy of your manuscript that highlights changes made to the original version. You should upload this as a separate file labeled 'Revised Manuscript with Track Changes'. - An unmarked version of your revised paper without tracked changes. You should upload this as a separate file labeled 'Manuscript'.   If you would like to make changes to your financial disclosure, please include your updated statement in your cover letter. Guidelines for resubmitting your figure files are available below the reviewer comments at the end of this letter.  If applicable, we recommend that you deposit your laboratory protocols in protocols.io to enhance the reproducibility of your results. Protocols.io assigns your protocol its own identifier (DOI) so that it can be cited independently in the future. For instructions see: https://journals.plos.org/plosone/s/submission-guidelines#loc-laboratory-protocols. Additionally, PLOS ONE offers an option for publishing peer-reviewed Lab Protocol articles, which describe protocols hosted on protocols.io. Read more information on sharing protocols at <https://plos.org/protocols?utm_medium=editorial-email&utm_source=authorletters&utm_campaign=protocols>.  We look forward to receiving your revised manuscript.  Kind regards,  Muhammad Nauman Ahmad, PhD  Academic Editor  PLOS ONE |  |
| --- | --- | --- |

**Point-by-point response**

**Reviewer #1:**

This study by Asghar et al., is about onion genotypes for resistance against purple blotch and management using different plant activators. The study is poorly planned, poorly written and the images are not clear to say it is A. porri.

**Response: Thank you very much for you constructive comments and suggestions. We have carefully revised the manuscript based on your feedback and made the following revisions:**

**-We have thoroughly reviewed and revised the manuscript to address concerns about its planning and writing. We have made significant improvements to enhance the overall clarity and consistency of the study.**

**-We have update the image of *A. porri* to improve their quality and clarity. The new image are higher resolution, and we have provided detailed figure legends to ensure accurate interpretation.**

Introduction:

-mention the crop loss in USD not percentages

**Response: Due to the lack of specific, accurate data on the economic losses caused by PBD worldwide, we are unable to provide precise figures. To address this, we have included a general statement in the manuscript highlighting the significant economic impact of PBD on onion crops.**

-use more references to talk about what management options are currently available to manage A.porri and what new the study is addressing.

**Response: We have updated the manuscript to include the references on current management option for *A. porri.***

M&M:

-use disease severity to explain the varietal response.

**Response: In present manuscript we use tha data of disease incidence, not disease severity. It is a reason that we cannot use the disease severity in the manuscript.**

-Disease control efficacy (%) = 100x[1-B/A]

**Response: We cannot use the disease control efficacy formula because we use the data of disease incidence after the application of plant defense activators against PBD.**

Results:

Remove table 2, 3. 4

**Response: It is necessary to understand the results and design of the experiment (Nested Structured Design), so it cannot be removed.**

Discussion:

-Use references to support/conclude the results you got.

**Response: We have revised the manuscript to include references that support and examine our results.**

-Read more literature to see how results are presented and manuscript is written. The image quality is poor.

**Response: We have improved the quality of the image and manuscript by adding latest references and quality of the language.**

-This work published in Plos one could be a good starting point (no conflict of interest to reviewer): https://www.ncbi.nlm.nih.gov/pmc/articles/PMC8775252/

**Response: Thank you for your proposal to read out the article, you have suggested. In the light of this article, we have improved our manuscript.**

**Thank you again for your valuable feedback. We hope that these revisions address your concerns and improve the quality of the manuscript.**

**Reviewer #2:**

The research article titled “Investigating Biochemical Variations in Onion Leaves Due to Purple Blotch Disease and its Management through Induced Resistance” reports the germplasm of onion against purple blotch disease, biochemical alteration analysis in onion due to pathogen invasion and evaluation of plant activators against purple blotch. This is a well-written article, and I anticipate that the manuscript should be of great interest to the researchers working on plant microbe interactions and pathogenicity. I considered the manuscript suitable for publication, subject to following improvements.

**Response: Many thanks for you constructive comments and suggestions to improve this MS!**

1. The abstract section should be more robust by adding some quantitative data on from the significant results.

**Response: We rewrote the abstract by adding the quantitative data.**

2. When a unique noun first appears, and you want to use the abbreviation later on then the full name must be accompanied by the abbreviation. Line 33 or 77: I suggest to add “PB” in the line 33.

**Response: We added “PB”.**

3. Line 41: “plant activators” add the names you evaluated.

**Response: We added all plant activators used in this study.**

4. Line 43: Replace “The contemporary study concluded” with “The present study revealed”.

**Response: We replaced.**

5. Revise the statement in Line 45-46: “While salicylic acid was proved to be highly significant in controlling the purple blotch in onion.”

**Response: We revised the statement.**

6. Merge paragraph No 4 from line 77-81 in the 3rd paragraph by adding a statement for coherence.

**Response: We merged the paragraph.**

7. Line 92: remove the word available and add the names.

**Response: We removed.**

8. Revise the objective section of your manuscript.

**Response: We revised the objectives in the manuscript.**

9. Line 97-98: A comprehensive survey was conducted in different onion growing areas of District Faisalabad. Add prominent names of the regions you collected samples.

**Response: We added.**

10. In line 104: the authors used surface sterilization for the samples and then they isolated the pathogens. It is confusing, did you isolated endophytic fungi? As you mentioned that the surface was sterilized. Justify and revise it.

**Response: We used surface sterilization to ensure only internal pathogens were isolated, not surface contaminants. This method confirmed the isolated fungi were responsible for the disease symptoms, not endophytic fungi.**

11. Revise the statement Line 117-118: The identification of pathogen was conducted on the basis of morphological and microscopic characters of the isolated pathogen.

**Response: We revised the statement.**

12. In results Line 217-219: How you confirmed the fungus at species level? All these phenotypic characters are common in different fungal species.

**Response: We confirmed the fungus at the species level through detailed morphological characterization, examining distinctive traits like conidia shape, size, and septation, as well as colony morphology.**

13. Cite latest references and improve the discussion section.

**Response: We cited latest references and discussion section improved.**

**Again Many Thanks!**

**Thank you to both the reviewers and the editor for your valuable feedback and thoughtful contributions to improving my manuscript.**

**Dr. Nasir Ahmed Rajput**
