# Supplementary material for: Investigating the biochemical variations in onion leaves due to purple blotch disease and its management through induced resistance
Source: PLoS One. 2025 May 9;20(5):e0323067. doi: 10.1371/journal.pone.0323067 (PMC12063818; doi:10.1371/journal.pone.0323067)
Supplement: Attachment — Submitted filename: Response to Reviewers.docx [file pone.0323067.s004.docx]

Dr. Nasir Ahmed Rajput

Associate Professor

Department of Plant Pathology,

University of Agriculture, Faisalabad Pakistan

Email: [nasirrajput81@gmail.com](mailto:nasirrajput81@gmail.com); [nasir.ahmed@uaf.edu.pk](mailto:rashid.iqbal@iub.edu.pk)

**Date: March 17, 2025**

**Dear Editor**

**PLOS ONE**

Many thanks to you and the both reviewers for your comments on our MS!

We have carefully revised our MS as requested by the reviewer #1 and #2 and formatted the MS file following the online instructions.

Please see the point-by-point response to the reviewers and the revised MS file for the details. We hope this revision is now satisfactory for publication.

Yours sincerely,

**Nasir Ahmed Rajput**

|  | Dear Dr. Rajput,  Thank you for submitting your manuscript to PLOS ONE. After careful consideration, we feel that it has merit but does not fully meet PLOS ONE’s publication criteria as it currently stands. Therefore, we invite you to submit a revised version of the manuscript that addresses the points raised during the review process.  Please submit your revised manuscript by Jan 22 2025 11:59PM. If you will need more time than this to complete your revisions, please reply to this message or contact the journal office at [plosone@plos.org](mailto:plosone@plos.org). When you're ready to submit your revision, log on to https://www.editorialmanager.com/pone/ and select the 'Submissions Needing Revision' folder to locate your manuscript file.  Please include the following items when submitting your revised manuscript:   - A rebuttal letter that responds to each point raised by the academic editor and reviewer(s). You should upload this letter as a separate file labeled 'Response to Reviewers'. - A marked-up copy of your manuscript that highlights changes made to the original version. You should upload this as a separate file labeled 'Revised Manuscript with Track Changes'. - An unmarked version of your revised paper without tracked changes. You should upload this as a separate file labeled 'Manuscript'.   If you would like to make changes to your financial disclosure, please include your updated statement in your cover letter. Guidelines for resubmitting your figure files are available below the reviewer comments at the end of this letter.  If applicable, we recommend that you deposit your laboratory protocols in protocols.io to enhance the reproducibility of your results. Protocols.io assigns your protocol its own identifier (DOI) so that it can be cited independently in the future. For instructions see: [https://journals.plos.org/plosone/s/submission-guidelines#loc-laboratory-protocols](https://track.editorialmanager.com/CL0/https:%2F%2Fjournals.plos.org%2Fplosone%2Fs%2Fsubmission-guidelines%23loc-laboratory-protocols/1/010f0193a85e00dc-a6958ebd-92b1-4e98-b36d-82151078494c-000000/hdhiLY88k1FJZgUjAQhBOiTyj5DTgeEXJHCan4dWCqQ=188). Additionally, PLOS ONE offers an option for publishing peer-reviewed Lab Protocol articles, which describe protocols hosted on protocols.io. Read more information on sharing protocols at [https://plos.org/protocols?utm_medium=editorial-email&utm_source=authorletters&utm_campaign=protocols](https://track.editorialmanager.com/CL0/https:%2F%2Fplos.org%2Fprotocols%3Futm_medium=editorial-email%26utm_source=authorletters%26utm_campaign=protocols/1/010f0193a85e00dc-a6958ebd-92b1-4e98-b36d-82151078494c-000000/oNrSHuXFhhnokzDiOkAE_yw01jtQcrpQFXykfZO_T_A=188).  We look forward to receiving your revised manuscript.  Kind regards,  Abhay K. Pandey Academic Editor PLOS ONE |  |
| --- | --- | --- |

**Point-by-point response**

**Reviewer #1:**

When evaluating the varieties for resistance - disease severity has to be taken into account. You have not collected the information. Your evaluation is flawed. A variety is resistant based on disease severity incidence the difference in disease severity. It is a open field experiment. Only disease incidence is not the appropriate measurement to say a variety is resistant.

Nanda et al., 2016 Identification of Novel Source of Resistance and Differential Response of Allium Genotypes to Purple Blotch Pathogen, *Alternaria porri* (Ellis) Ciferri (no conflict of interest to reviewer)(<https://www.ncbi.nlm.nih.gov/pmc/articles/PMC5117860/pdf/ppj-32-519.pdf>) - used disease incidence and disease severity to identify disease resistant variety. Expt was also conducted in invitro condition to evaluate it.

Response: Thank you very much for your suggestions and improvements. We have re-conducted our experiment after obtaining additional time from the editor for revision. As per your suggestion, we have added new data on disease severity instead of disease incidence.

--------------------------------------------------------------------------------------------------------------

Specific comments:

1). Line 73,75, 231, 241, 301, 380, 448, 460 - space between words

Response: All typo mistakes has been acknowledged.

--------------------------------------------------------------------------------------------------------------
2). Line 135 – specimen

Response: Corrected
--------------------------------------------------------------------------------------------------------------

3). Line 143 - how many days?

Response: Added as “The fungal pieces were transferred to new sterilized petri plates containing fresh potato dextrose agar (PDA) and incubated at 25°±5°C for seven days.”
--------------------------------------------------------------------------------------------------------------
4). L161 - Is the location chosen for the expt is a hotspot for PBD?

Response: Yes, PBD was frequently observed at the location (Vegetables' Research Area, Ayub Agriculture Research Institute (AARI), Faisalabad) of experiments
--------------------------------------------------------------------------------------------------------------
5). L196-Separate

Response: Corrected
--------------------------------------------------------------------------------------------------------------
6). L301- recorded

Response: Corrected
--------------------------------------------------------------------------------------------------------------
7). Table 2&3 , Line 293, 314 - expand SOD - titles should be stand alone

Response: Corrections has been incorporated
--------------------------------------------------------------------------------------------------------------
8). Line 322 – recorded

**Response:** corrected
--------------------------------------------------------------------------------------------------------------
9). Table 4, 5 - expand PoD

**Response:** We have added full names.
--------------------------------------------------------------------------------------------------------------
10). L334, Table 6&7, Figure 6 - expand CAT

**Response:** Acknowledged in tables and fig.
--------------------------------------------------------------------------------------------------------------

**Reviewer #2:**

1). The statistical analysis section is not added to the revised manuscript.
It is suggested to add a statistical analysis section in the material and methods.

**Response: The data related to statistical analysis in methodology section has been added.**
--------------------------------------------------------------------------------------------------------------

**Thank you to both the reviewers and the editor for your valuable feedback and thoughtful contributions to improving my manuscript.**

**Dr. Nasir Ahmed Rajput**
